# Imaging of aortic coarctation using Gd-DTPA and Gadofosveset: a comparative study

**DOI:** 10.1186/1532-429X-11-S1-P261

**Published:** 2009-01-28

**Authors:** Gerald F Greil, Marcus R Makowski, Andrea J Wiethoff, Vicky Parish, Sergio Uribe, Christian Jansen, Aaron Bell, Phillip Beerbaum, Martin Rohrer, Rene M Botnar, Reza Razavi, Tobias Schaeffter

**Affiliations:** 1 Imaging Sciences Division, King's College London, St Thomas' Hospital, London, UK; 2 European Business Unit Diagnostic Imaging, Bayer Schering Pharma AG, Berlin, Germany

**Keywords:** Aortic Arch, Inversion Recovery, Stent Implantation, Aortic Coarctation, Free Breathing

## Objective

The use of Gadofosveset in combination with a 32 channel coil and optimized image sequences allows high resolution free breathing and ECG triggered imaging of the aortic arch in patients with coarctation with improved imaging results compared to previous techniques.

## Background

First-pass breath-hold non-ECG-triggered 3D contrast-enhanced-magnetic-resonance-angiography (CEMRA) using Gd-DTPA is commonly used for assessment of the aortic arch. However, image resolution is limited due to time constraints and vascular borders are blurred due to vascular motion and insufficient breath holds.

## Methods

In 7 patients (30 **±** 7 yrs) the aortic arch was imaged after surgical repair (n = 6) or stent implantation (n = 1) on a 1.5 T clinical scanner (Philips Medical Systems). Patients were investigated twice within 7 days using Gd-DTPA (day 1, 0.10–0.17 mmol/kg) and Gadofosveset (day 2, 0.03 mmol/kg). First pass breath hold 3D CEMRA as well as a respiratory navigator gated and end-diastolic ECG triggered 3D steady-state free precession (SSFP) sequence with a T2 prepulse were used. Gadofosveset allowed the application of an inversion recovery (IR) prepulse to suppress surrounding tissue signal. Results were compared (Table 1).

## Results

**Figure 1:**
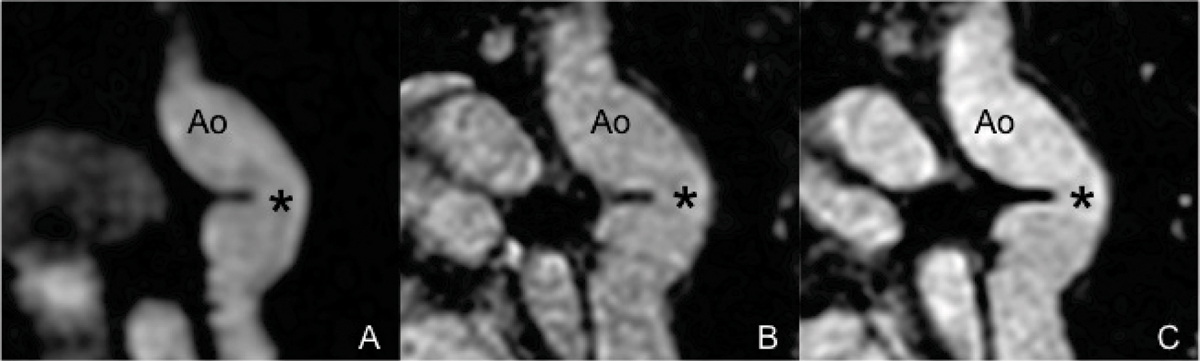
Figure 1

The navigator gated and ECG triggered 3D IR SSFP (Figure 1C) sequence showed best image quality results (Table 1). Cross sectional areas showed good interstudy agreement in the 3D SSFP technique without IR (Figure 1B) and 3D first pass CEMRA (Figure 1A) with similar image quality results using Gadofosveset and Gd-DTPA. However, these areas are smaller in end-diastolic ECG triggered respiratory gated sequences with and without IR prepulse than in breath hold 3D CEMRA (Table 1, all p < 0.05). Stent artifacts were similar in all sequences.

**Table 1:** Values are expressed as mean ± standard deviation

Contrast Agent	Sequence	Contrast to Noise Ratio (CNR)	Vessel Wall sharpness (%)	Image quality (mean ± SD)	Vessel Area (cm^2^)	Isotropic spatial resolution (mm^3^)
Gd-DTPA	CEMRA	110 ± 10^†^	41 ± 4^†^	2.4 ± 0.8^†^	4.6 ± 1.9^†^	1.77
	SSFP	135 ± 11*	48 ± 6*	3.3 ± 0.5*	4.1 ± 1.7*	1.49
Gadofosveset	CEMRA	99 ± 21^†^	40 ± 4^†^	2.7 ± 0.5^†^	4.7 ± 2.1^†^	1.77
	SSFP	128 ± 19*	46 ± 3*	3.1 ± 0.7*	4.1 ± 1.6*	1.49
	SSFP+IR	154 ± 14	53 ± 5	3.7 ± 0.5	3.9 ± 1.7	1.49

^†^*, no significant differences between corresponding sequences (Gd-DTPA vs Gadofosveset). Image quality: 1 = non diagnostic, 2 = diagnostic, 3 = good, 4 = excellent.

## Conclusion

A respiratory-navigator-gated and ECG-triggered 3D-IR-SSFP-sequence after application of Gadofosveset allows free-breathing end-diastolic high-resolution imaging of the aortic arch in combination with a 32-channel-coil. Image quality is superior with slightly smaller cross sectional areas compared to first-pass CE-MRA.

